# A prospective cohort study investigating contributors to mild cognitive impairment in adults with spinal cord injury: study protocol

**DOI:** 10.1186/s12883-020-01899-7

**Published:** 2020-09-11

**Authors:** Danielle Sandalic, Ashley Craig, Mohit Arora, Ilaria Pozzato, Grahame Simpson, Bamini Gopinath, Jasbeer Kaur, Sachin Shetty, Gerard Weber, Ian Cameron, Yvonne Tran, James Middleton

**Affiliations:** 1 John Walsh Centre Rehabilitation Research, Northern Clinical School, Faculty of Medicine and Health, The University of Sydney, Kolling Institute, Royal North Shore Hospital, St Leonards, NSW 2065 Australia; 2 Royal North Shore Hospital, St Leonards, NSW 2065 Australia; 3 Prince of Wales Hospital, Randwick, NSW 2031 Australia; 4 Royal Rehab, Ryde, NSW 2112 Australia; 5 Australian Institute of Health Innovation, Macquarie University, North Ryde, NSW 2113 Australia

**Keywords:** Brain injuries, Cognition disorders, Cognitive dysfunction, Mild cognitive impairment, Mood disorder, Nervous system injury, Spinal cord injuries

## Abstract

**Background:**

Studies report rates of mild cognitive impairment (MCI) in spinal cord injury (SCI) range between 10 and 60%. This broad estimate of MCI in SCI is most likely a result of: (i) inconsistent operationalization of MCI; (ii) heterogeneity among individuals with SCI; (iii) failure to account for MCI subtypes, thereby adding to the heterogeneity of samples; and, (iv) poor control for traumatic brain injury (TBI) that obscures differentiation of MCI attributable to TBI versus other factors. There is a paucity of longitudinal studies following the course of MCI in SCI, and none that account for multiple predictors of MCI, including interactions among predictors.

**Methods:**

An inception cohort longitudinal study will assess approximately 100 individuals aged 17–80 years with acute SCI, with measures taken at three timepoints (baseline, 3 months post-baseline, and 12 months post-injury). Data relevant to medical care received within the first 24–48 h of presentation to the emergency department will be analysed, as will measures of cognition, injury characteristics, medical history, personal factors, psychological status, psychosocial functioning, and quality of life. Latent class mixture modelling will determine trajectories for the primary outcome of interest, cognitive functioning and its subtypes, and secondary outcomes of interest such as depression. Multiple regression analyses will identify predictors of MCI and its subtypes.

**Discussion:**

The prospective design will reveal change in cognitive functioning across time and unveil different outcome trajectories; thus addressing the lack of knowledge on trajectories of MCI and MCI subtypes in SCI. Through subtyping MCI, we hope to yield groups of cognitively impaired individuals with SCI that are potentially more homogenous and thereby stable and predictable. This is the first study to capture emergency department and acute care diagnostic evidence of mild TBI, which has been poorly controlled in previous studies. Our study will also be the first to distinguish the contribution of TBI from other factors to the development of MCI in individuals with SCI.

**Trial registration:**

The study was prospectively registered with the Australian and New Zealand Clinical Trial Registry (ACTRN12619001702101) on 3rd December 2019.

## Background

Spinal cord injury (SCI) occurs as the result of damage to the nervous system and spinal cord, associated with bruising or severing in traumatic injuries [1], or to degeneration of the spine, infections, vascular accidents, or cancerous tumours in non-traumatic injuries [2]. SCI is associated with a range of secondary health conditions, including; sleep disturbance [3, 4], psychological disorder [5], chronic pain [6], fatigue [7, 8], and autonomic nervous system dysfunction [9, 10].

Cognitive impairment can be a significant problem following SCI, with a recent systematic review indicating the occurrence of cognitive impairment among individuals with SCI is in the range of 10 and 60% [11]. In line with this, it has been proposed that individuals with SCI demonstrate an accelerated cognitive aging process [12, 13], to the extent that they may be 13 times more susceptible to cognitive impairment when compared to able-bodied individuals [14]. Studies in people with SCI have mostly investigated mild cognitive impairment (MCI), a form of impairment that is defined by subjective impairments that are objectively verified without serious impairments in functioning [15–19], raising questions regarding the clinical significance of cognitive impairment for SCI rehabilitation outcomes.

A number of issues have contributed to confusion regarding the definition and nature of cognitive impairment as it relates to SCI, with varying operationalisations of the construct making it difficult to confirm the severity and hence clinical relevance of cognitive impairment in SCI [11, 14]. These issues which limit understanding of the effects of MCI on outcomes following SCI are discussed below:

First, objective criteria for the confirmation of an impairment have varied greatly across SCI studies with respect to the stringency of criteria that have been applied and the types of tests utilized in the assessment of impairments [11]. It is therefore difficult to compare studies given the incorporation of different measures (e.g., scores on the Neuropsychiatry Unit Cognitive Assessment Tool [14] versus Rey Word Recognition Test [20] versus Symbol Digit Modalities Test [21]). Some studies have failed to pre-specify criteria for impairment altogether leading to reliance on significant between-group differences as proxies for impairment [12, 13]. Standard deviations (SD) from normal cognitive functioning that signify cognitive decline have ranged from 1 to 2 SD from the mean [14, 22]. Outside the SCI literature, a − 1.5 SD from normal in a single cognitive domain identifies the largest number of MCI diagnoses, compared to a − 2.0 SD in one or more cognitive domains which identifies the least [23].

Second, heterogeneity among participants has limited the predictive validity of the MCI diagnosis [11]. Individuals with SCI display different trajectories of MCI, varying from remission [21, 24], stabilization [25], to worsening [13], possibly as a result of distinct underlying ‘causes’ that can be identified as differentiable etiological subtypes [26, 27]. It has been suggested that the predictive validity of a MCI diagnosis improves by subtyping [28], but as far as known, there are no SCI studies that have applied subtyping to clarify such distinctions.

Third, most earlier studies have concentrated on traumatic brain injury (TBI) as the main cause of MCI, occurring before SCI, or at the time of SCI [22, 24, 29–37]. There are, however, many factors that contribute to the development of MCI after SCI [11]. These may include functional changes to the cardiovascular [38–40] and autonomic nervous system [41], psychological changes such as mood disturbances [42], medications [43–45], ageing [14, 46–48], sleep disorder [49, 50], fatigue [8], and social changes, such as the potential for decreased social participation [51, 52]. Some of these risks (e.g., depression, polypharmacy) are transient, while others (e.g., brain injury, cardiovascular dysfunction) contribute to degenerative or long-term courses of cognitive impairment. Therefore, it makes sense that MCI manifests differentially across individuals with SCI, who despite having SCI in common, often present with variable SCI-related complications indicative of different ‘risk profiles’. To study MCI in individuals with SCI it is important to apply risk-profiling to yield homogenous groups and to observe the time-course of risks independently and as a series of interactions. There are very few if any studies that fulfil this agenda.

As stated above, there are various factors associated with MCI after SCI. Autonomic nervous system disturbances and associated cardiovascular and cerebrovascular complications following SCI have been studied as risks for the development of cognitive impairment and poor SCI-related outcomes [38, 41, 53]. All individuals with SCI above the sixth thoracic segment (T6) suffer decentralised cardiovascular control, which has been found to predict cerebral hypoperfusion and consequently impaired cognitive performance [53]. Individuals with paraplegia can suffer persistent elevations in heart rate and related arterial stiffness, and this has been shown to predict suboptimal cognitive performance [54]. Measures of autonomic balance (measured by heart rate variability in this study), blood pressure, and heart rate, could be incorporated with other risk measures to generate risk profiles of the likelihood a person with SCI will develop vascular forms of MCI and guide monitoring and follow-up cognitive assessments.

TBI significantly increases an individual’s risk of MCI after SCI [11, 13, 18–26]. Mild and mild-complicated forms of TBI interfere less with cognition than do severe forms of TBI; however, mild-complicated and moderate TBI appear undifferentiable in terms of their influence on cognition [35, 55, 56]. Mild TBI is believed to account for up to 90% of TBI [57], with 10% being mild-complicated TBI [58] meaning there is evidence of intracranial lesions on structured neuroimaging techniques [59, 60]. Research concerning the relationship between SCI and TBI has failed to separate mild from other forms of TBI [32], possibly due to challenges in the acute identification of mild injuries (e.g., loss of consciousness assessed within 30 min of injury or amnesia assessed within 24 h post-injury). The difficulty of tracking people with mild TBI is exemplified by a study by Powell et al. [61] where the diagnosis of mild TBI in a non-SCI sample was missing in emergency department medical records in over 50% of individuals meeting Centers for Disease Control and Prevention criteria for mild TBI.

Research into medication-use in the rehabilitation of SCI and its relationship to the development of cognitive impairment is similarly inconclusive. Recently, Krebs et al. [43] found no relationship between antimuscarinic treatment of neurogenic lower urinary tract dysfunction and subsequent performance on neuropsychological testing after a treatment-period of 3 months. Two studies independently exploring the effects of anticholinergic burden and gabapentin on cognition found limited evidence of adverse reactions relating to the use of these medications in patients with SCI [44, 45]. These results are in contrast to a sizable body of evidence of negative associations between anticholinergic medication-use and cognitive impairment in able-bodied individuals [62–66] and individuals comprising other illness samples [67–71], and may relate to inadequate length of follow-up, insufficient statistical power to detect effects, and/or use of measures that lack sensitivity to detect cognitive decline.

Support for a relationship between MCI and depression in SCI has been found in three of six relevant studies [5, 8, 72]; however, lack of control for the effects of TBI makes it difficult to rule-out the contribution of TBI to this relationship. Major depressive disorder symptoms include difficulties with concentration and/or decision-making [73], so it is conceivable that depression contributes to MCI in some individuals with SCI, at least for the duration of a reactive depressive episode. Where depression is related to endogenous ‘causes’ as in the case of late-life depression or vascular depression [74], depression has predicted the conversion of MCI to Alzheimer’s disease in elderly able-bodied samples [42], suggesting that there is a strong link between cognitive decline and depression, where depression is biologically driven. Clearly, predictors such as depression possess heterogeneity that should be considered when exploring their relationship to the development of MCI and its course.

Finally, the impact of moderators of relationships between predictors of MCI and MCI need to be considered, and in this respect, attention must be given to age as a potential moderator of relationships. Four of five studies [14, 46–48] into the impact of age on risks associated with cognitive impairment following SCI have suggested that age compounds risks associated with cognitive impairment following SCI. This is significant given the mean age of new-onset SCI has increased since earlier studies into the relationship between cognitive impairment and SCI were conducted [75, 76].

In response to the aforementioned issues and with the intention of addressing the limitations of prior studies undertaken on the topic, we present a protocol for an inception cohort longitudinal study that will investigate factors contributing to cognitive impairment in individuals with SCI across a 12-month period with the incorporation of controls for the effects of TBI (i.e., non-TBI/cognitively impaired persons with SCI). We outline the study’s design and method below, including procedures for participant recruitment and allocation to groups, predictor and outcome measures, data collection, and statistical analyses.

## Methods

### Primary objectives

- Determine the rate of MCI in adults with SCI, including MCI sub-types: single domain amnestic, multiple domain amnestic, single domain non-amnestic, and multiple domain non-amnestic;
- Identify predictors of MCI (e.g., mood disturbance, polypharmacy, presence of TBI) and moderators of relationships between predictors and MCI (e.g., education, estimated premorbid intelligence, practice effects);
- Investigate how predictors operate over time to influence manifestations and trajectories of MCI (e.g., how depression and/or polypharmacy affect MCI).

### Secondary objectives

- Compare adults with SCI with and without MCI against a range of psychosocial and secondary health outcomes (e.g., depression, health complications, quality of life).

### Study design

An inception cohort longitudinal study will follow adults with SCI from the first 24–48 h of their presentation to the emergency department. The primary outcome cognition and its predictors and secondary outcomes will be measured across rehabilitation and discharge up to 12-months post-injury.

### Settings

Participants will be recruited from two major acute-care SCI units in New South Wales, Australia; namely, Prince of Wales Hospital and Royal North Shore Hospital from December 2019 through December 2021. Subsequently, they will be followed over the course of their rehabilitation at a specialised rehabilitation centre; i.e., Royal Rehab or Prince of Wales Hospital, until discharge. The 12-month post-injury follow-up assessments will be conducted online when participants are living in the community.

### Participants

#### Inclusion criteria

- Aged 17–80 years inclusive with acute SCI from non-traumatic or traumatic etiology;

#### Exclusion criteria

- Insufficient proficiency in English language;
- Severe mental (e.g., schizophrenia) and/or physical illness, including severe TBI.

### Participant allocation to groups

**Fig. 1:**
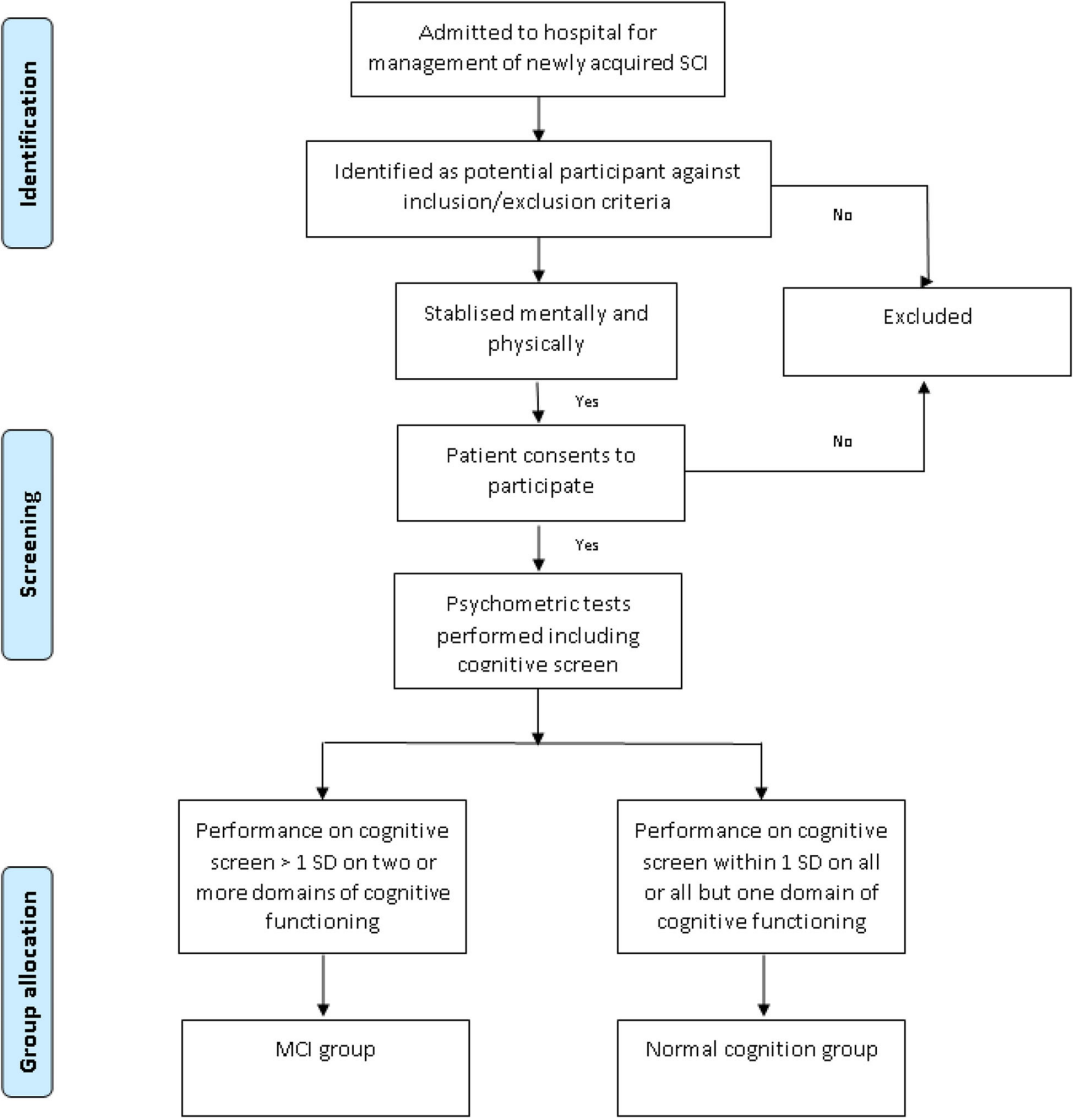
Participant allocation to groups about here

Allocation to groups will be based on the absence or presence of cognitive impairment as presented in Fig. 1. Cognitive performance will be assessed using a validated neuropsychological screen. The number of participants will be up to 50 per group, and recruitment will be stratified so that similar numbers of females/males and tetraplegia/paraplegia are allocated to groups. The criteria for a diagnosis of MCI, and hence allocation to cognitively impaired versus not-cognitively impaired groups, will accord with those proposed by Jak et al. [77], requiring scores > 1 standard deviation below normal on ≥2 tests within a cognitive domain on a standardised neuropsychological test. These criteria have been selected as they have demonstrated greater stability over time when compared to alternative conventional criteria as proposed by Petersen, that require scores of > 1.5 standard deviations below normal on a single test within one cognitive domain on neuropsychological testing. We will subtype MCI as follows: *Single Domain Amnestic* if only the memory domain is impaired; *Single Domain Non-Amnestic* if only one non-memory domain is impaired; *Multiple Domain Amnestic* if memory and at least one other domain shows impairment; and *Multiple Domain Non-Amnestic* if there are impairments in more than one non-memory domain [78].

### Variables

Table 1 shows predictors, primary and secondary outcome measures, and the assessment timepoints relating to each measure. As the table indicates, predictors are marked by a single asterisk whereas secondary outcomes are marked by a double asterisk. *Acute monitoring* (V0) refers to the time from first presentation to the emergency department to the time of consent; at which point, routinely collected data will be retrospectively retrieved from medical records. *At rehabilitation* (V1) may vary between five and 12 weeks following SCI as participants will vary with respect to the amount of time required spent in acute care prior to proceeding to rehabilitation. *Discharge* (V2) refers to a three- to four-month period following assessments completed at V1. The timepoint *in community* refers to 12-months after the date of the initial SCI.

**Table 1:** Predictors, primary and secondary outcome measures, and assessment timepoints

Assessment time-points	Visit-type				
Study phase	Acute monitoring	At rehab		At discharge	In community
Visit number		(V0)	(V1)	(V2)	(V3)
Eligibility pre-screening (by treating Clinician)		✓			
Informed Consent			✓		
**Primary outcome**					
**Measure**	**Measurement tool**				
Cognitive functioning	NUCOG		✓	✓	✓
Predictors* and secondary outcomes**					
**Measure**	**Measurement tool**				
Autonomic assessment*	HRV		✓	✓	✓
Current medications*	Medical records	✓	✓	✓	✓
Diagnostic evidence of TBI*	Cerebral CT/MRI scan	✓			
	GCS	✓			
	PTA	✓			
Demographics*	Medical records	✓			
Environmental factors*	Carer/family status		✓	✓	✓
	Compensation status		✓	✓	✓
Injury characteristics*	Medical records	✓			
Medical History*	Medical records	✓			
Other co-morbidities	Medical records*	✓			
	SCI SCS**		✓	✓	✓
Participation measure**	WHODAS domain				✓
Personal factors**	ADAPSS		✓	✓	✓
	Brief COPE		✓	✓	✓
	CD-RISC Short Form		✓	✓	✓
	MSES		✓	✓	✓
	WHO ASSIST		✓	✓	✓
Psychological measures**	GAD-7		✓	✓	✓
	PCL-5		✓	✓	✓
	PHQ-9		✓	✓	✓
Psychosocial measures**	Berlin Questionnaire		✓	✓	✓
	FSS		✓	✓	✓
	ISCoS pain dataset		✓	✓	✓
	PCS		✓	✓	✓
	PSQA		✓	✓	✓
	SSQ		✓	✓	✓
QoL measure**	EQ-5D-5L		✓	✓	✓
Return to work**	Questionnaire				✓
Severity of injury*	SCI: AIS and FIM	✓			
	TBI: WPTAS	✓			
Transition ED*	ED record	✓			
Vital signs*	BP, HR, RR, and Temp	✓	✓	✓	✓

*Abbreviations*: *ADAPSS* Appraisal of DisAbility Primary and Secondary Scale, *AIS* American Spinal Injury Association Impairment Scale, *BP* blood pressure, *Brief COPE* Coping Orientation to Problems Experienced, *CD-RISC* Connor-Davidson Resilience Scale, *ED* Emergency Department, *EQ-5D-5L* Euro Quality of Life 5-Dimensional 5-level, *FIM* Functional Impairment Scale, *FSS* Fatigue Severity Scale, *GAD-7* Generalized Anxiety Disorder 7-Item Scale, *GCS* Glasgow Coma Scale, *HR* heart rate, *HRV* heart rate variability, *ISCoS* International Spinal Cord Society, *MH* Mental health, *MSES* Moorong Self-efficacy Scale, *NUCOG* Neuropsychiatry Unit Cognitive Assessment Tool, *PCS* Pain Catastrophizing Scale, *PHQ-9* Patient Health Questionnaire-9, *PSQA* Pittsburgh Sleep Quality Assessment, *PTA* post-traumatic amnesia, *PCL-5* Post-traumatic Stress Disorder Checklist for the Diagnostic and Statistical Manual of Mental Disorders Firth Version, *RR* respiratory rate, *SCI* spinal cord injury, *SCI SCS* Spinal Cord Injury Secondary Condition Scale, *SSQ* Social Support Questionnaire, *TBI* traumatic brain injury, *Temp* temperature, *WHO ASSIST* World Health Organisation Alcohol Smoking and Substance Involvement Screening Test, *WHODAS* World Health Organisation Disability Assessment Scale, *WPTAS* Westmead Post Traumatic Amnesia Scale

*Predictor measure

**Outcome measure

### Measurement

#### Mode of assessment

Participants will be assessed either face-to-face or via a secure online platform called Research Electronic Data Capture (REDCap) [79]. Participants entering their responses online are given their personal REDCap link and instructions on how to complete.

#### Primary outcome measure

##### Neurocognitive testing

The Neuropsychiatry Unit Cognitive Assessment Tool (NUCOG) [80], a validated cognitive measure for SCI, will be used to screen cognitive functioning using age-adjusted normative scores provided in the administration and scoring manual to identify deviations from normal cognitive functioning. Means and SD for each of the five subscales comprising the NUCOG (attention, visuoconstructional, memory, executive, and language) permit analyses of patterns of performance across subscales. The tasks presented in each of the subscales are listed below:

Attention: Measures of orientation, forward and backward digit span, and the recitation of an overlearned sequence.

Visuoconstructional: Drawing reproduction, orobuccal and limb praxis, left-right orientation, spatially-directed attention, and calculation.

Memory: Verbal recall of words, spatial recall through the re-drawing of previously copied figures, autobiographical and other personal or historical details.

Executive function: Motor sequencing, categorical/semantic fluency, abstract thinking, and interference.

Language: Comprehension, repetition, naming, writing, reading, and word-finding.

Items requiring normal hand function (e.g., drawing reproduction) will be adapted as per previous studies where this has been shown to not alter the validity of NUCOG scores [81]. Where it is not possible to administer the NUCOG face-to-face (e.g., discharge to the community outside metropolitan areas within the state of NSW, Australia, or social distancing restrictions due to COVID-19), administration will occur via telehealth methods. Administration of the NUCOG via teleconferencing required further adjustments to the NUCOG assessment procedure to suit the telehealth environment. These procedural adjustments will be standardized, and validation will constitute part of this study.

#### Secondary measures

##### Autonomic assessment

The assessment of heart rate variability (HRV) will be incorporated into analyses of the influence of cardiovascular dysfunction on MCI. This will involve a Biosemi Active-Two System, 32 channels, at 2480 Hz sampling rate to obtain three-lead electrocardiograph recordings with Ag/AgCl electrodes positioned according to Einthoven’s triangle (beneath the right and left clavicles, and on the lower edge of left rib cage on the anterior axillary line). Additional channels will measure respiration rate (sensitive band around the chest), and skin conductance (surface electrodes on second and fourth fingers of non-dominant hand), which will be collected as indicators of sympathetic nervous system activity.

##### TBI screening

TBI will be identified at the time of presentation to the emergency department and throughout the period of medical care using the following indicators: (i) Glasgow Coma Scale scores at the time of injury and at the time of presentation to the emergency department; (ii) evidence of intracranial injuries from medical imaging; (iv) periods of post-traumatic amnesia (PTA) as assessed by the Abbreviated Westmead Post-traumatic Amnesia Scale (A-WPTAS), and/or, (v) reported periods of disorientation, confusion and/or witnessed periods of unconsciousness that is not accounted for by delirium or substance withdrawal. Additionally, participants will be interviewed at the time consent is obtained with proxy questions assessing the presence of any self-report TBI indicators that were evident at the time of injury, to corroborate diagnostic evidence available from medical records. A probabilistic approach will be used to identify the occurrence of TBI by cross-checking clinically-documented evidence of TBI against self-reported information relevant to TBI [82].

##### Self-report questionnaires

*Generalised anxiety disorder-7 (GAD-7)* [83] GAD-7 is a seven-item instrument that is used to assess the severity of generalized anxiety disorder (GAD). Items ask individuals to rate the severity of symptoms experienced over the past 2 weeks. Responses including ‘not at all,’ ‘several days,’ ‘more than half the days,’ and ‘nearly every day’ are summed to provide a total score. Total scores of 5, 10, and 15 respectively represent cut-points for mild, moderate, and severe GAD symptoms.

*Patient health questionnaire-9 (PHQ-9)* [84] PHQ-9 asks respondents to rate each of the nine Diagnostic and Statistical Manual of Mental Disorders Fourth Edition (DSM-IV) criteria for depression over a two-week timeframe. Ratings for items ranging from 0 ‘not at all’ to 3 ‘nearly every day’ are summed to determine depressive-symptom severity (0–4 no depressive symptoms; 5–9 mild depressive symptoms; 10–14 moderate depressive symptoms; 15–19 severe depressive symptoms; and 20–27 severe depressive symptoms).

*Post-traumatic stress disorder checklist for the diagnostic and statistical manual of mental disorders version V short-form (PCL-5-SF)* [85] PCL-5-SF is a 4-item abbreviation of the full 20-item PCL-5 that has been shown to reliably screen for symptoms of post-traumatic stress disorder (PTSD) [86]. Patients are asked to rate symptoms they have experienced over the past month including re-experiencing symptoms, symptoms of avoidance, negative alterations in cognition and mood, and alterations in arousal and reactivity on a 5-point Likert-type scale ranging from ‘not at all’ to ‘extremely’ often. Scores indicating a likely diagnosis of PTSD vary in accordance with the desired level of sensitivity and specificity; conservative (PCL-5 ≥ 38), liberal (PCL-5 ≥ 28), or intermediate (PCL-5 ≥ 32 or DSM-5 Criteria B-E) [85].

*Adapted version of the brief pain inventory (BPI)* [87] Three items from the Interference scale of the full Brief Pain Inventory (BPI) and one item from the severity scale of the BPI will be included to capture patients’ pain experiences. The three interference items included are those selected for inclusion in the International Spinal Cord Injury Basic Pain Data Set Version 2.0; ‘how much has your pain interfered with your: activities, mood, and night’s sleep in the last week,’ with response options ranging from 0 ‘no interference’ to 10 ‘extreme interference.’

*Social support questionnaire short form (SSQ6)* [88] SSQ-6 is a 6-item measure of social support that asks patients to indicate the number of people available to them for the provision of support, and to rate how satisfied they are with the level of support they receive. Satisfaction ratings range from ‘very dissatisfied’ to ‘very satisfied’ on a 6-point Likert-type scale.

*Pain catastrophizing scale (PCS)* [89] PCS is a self-report cognitive bias measure consisting of 13 items that screen for the presence of catastrophizing thoughts (e.g., “I can’t stop thinking about how much it hurts”) scored 0 to 4 resulting in a total possible score of 54. Scores above 30 indicate clinically significant levels of pain catastrophizing.

*Fatigue severity scale (FSS)* [90] FSS is a 9-item self-report scale that measures the severity of fatigue and its effects on activities and lifestyle. Items are scored on a 7-point scale Likert-type scale (1 = strongly disagree and 7 = strongly agree). The higher the score the more severe the fatigue.

*The Pittsburgh sleep quality index (PSQI)* [91] PSQI measures the quality and patterns of sleep in seven domains: subjective sleep quality, sleep latency, sleep duration, habitual sleep efficiency, sleep disturbances, use of sleep medication, and daytime dysfunction. Scoring of items is based on a 0 to 3 Likert scale with a score of 3 reflecting the negative extreme. Scores greater than a total of 5 indicate poor sleep.

*Spinal cord injury secondary conditions scale (SCISCS)* [92] SCISCS assesses secondary physiological conditions that are associated with SCI using a 4-point ordinal scale ranging from 0 (not experienced/insignificant problem) to 3 (significant/chronic problem). Participants are asked to rate the degree to which each of the 16 items have affected their activities and independence in the last 3 months. Examples of secondary conditions include pressure ulcers and bladder dysfunction. Total scores range from 0 to 48 with higher scores indicating greater overall problems with secondary physiological conditions.

*World Health Organisation alcohol, smoking, and substance involvement screening test (WHO ASSIST)* [93] Question 2 of the WHO ASSIST which asks about frequency of use of each of 10 substances (tobacco, alcohol, cannabis, cocaine, amphetamine-type stimulants, inhalants, sedatives, hallucinogens, opioids, and ‘other’ drugs) during the past 3 months is included as this provides critical information about the substances most relevant to current health status. Responses to this question are rated on a 5-point frequency scale ranging from ‘never’ to ‘daily or almost daily.’

*Brief coping orientation to problems experienced (brief COPE)* [94] Brief COPE is a 28-item self-report questionnaire that measures effective and ineffective ways to cope with stressful life events including reactions to serious medical diagnoses. Only questions 2, 3, 7, and 8 of the Brief COPE are included to minimise time of testing. These four questions permit calculation of scores for the ‘Active – Approach’ and ‘Avoidant – Denial’ scales.

*Moorong self-efficacy scale (MSES)* [95] MSES was developed to measure self-efficacy as it relates to the performance of functional activities of daily living in individuals with SCI. Participants rate their confidence in their ability to complete 16 tasks on a 7-point Likert-type scale with 1 = very uncertain and 7 = very certain. The higher the score, the greater perceived self-efficacy to perform functional activities.

*Connor-Davidson resilience scale (CDRS)* [96] CDRS is a 25- item self-report measure of personal resilience with higher scores reflecting greater resilience. Only questions 1 ‘able to adapt to change’ and 8 ‘tend to bounce back after illness or hardship’ are included to appraise patients’ coping over the past month or the period of acute adjustment to SCI and hospitalisation. This two-item CDRS has demonstrated internal consistency, test-retest reliability, convergent validity, and divergent validity, as well as significant correlations with the full scale [96].

Appraisals of DisAbility: Primary and Secondary Scale (ADAPSS) [97] Short-Form.

ADAPSS Short-Form is measure of SCI-specific appraisals, with a 2-factor structure of catastrophic negativity and determined resilience.

*EUROQOL version 5D-5L (EQ-5D-5L)* [98] EQ-5D-5L comprises five dimensions each describing different aspects of health: mobility, self-care, usual activities, pain/discomfort, and anxiety/depression. Each dimension has three response levels: no problems, some problems, extreme problems. Participants are asked to indicate their health status by checking the most appropriate response-level for each of the five dimensions. The digits for the five dimensions can be combined into a 5-digit number yielding a description of health.

### Bias

Several processes will help to reduce bias in the analyses. First, only adults with a new SCI will be recruited into the study. Second, inclusion and exclusion criteria will be strictly adhered to reduce unwanted confounding of data. Third, medical information such as PTA testing undertaken in the first 24 to 48 h after the SCI that informs the presence of mild TBI, as well as other relevant measures and TBI proxies, will be harvested from medical records to confirm the presence of a mild TBI, permitting the differentiation of TBI from other predictors of MCI. Fourth, to minimize loss to follow-up, multiple options for assessment will be provided, including online assessment, face-to face paper assessment, teleconference, and telephone assessment. Follow-up reminders by email or telephone texts will be incorporated to enhance adherence to study participation. Fifth, the use of linear mixed model repeated measures will reduce the impact of loss of missing data.

### Study size

Our preliminary study of 150 adults with SCI [14] found a 10-point difference in the NUCOzG total mean scores (with a SD of 6.3) between the participants with MCI (*n* = 60) versus those without (*n* = 90). Therefore, the resultant effect size was large being > 1.5. For this prospective study, to test the primary outcome of interest, we are conservatively assuming a between-groups moderate Cohen’s d effect size of 0.7, a group allocation of 0.6:1 MCI: non-MCI, an alpha of 0.05, 80% statistical power, three assessment points, a compound symmetry correlation matrix (rho = 0.5), and attrition rates of 20 and 30% between assessment points. The estimated required overall sample size is at least 90 participants. This calculation was based on linear mixed model of repeated measures with a general correlation structure and the estimated sample size includes limited interaction effects [99].

### Statistical methods

Data analyses will be performed using SPSS v.25. Descriptive statistics will be generated for all relevant primary and secondary outcomes at all time-points. Multiple regression analyses will be used to identify predictors of MCI at discharge and at 12-months post-injury for the total sample and for all subgroup analyses. Latent class mixture modelling will be used to determine trajectories for different domains of cognitive functioning, as well as for selected secondary measures like anxiety, depressive mood and quality of life. Repeated measures linear mixed model analyses will be used to determine differences between the cognitive impairment versus no cognitive impairment groups for secondary outcomes. All analyses will be adjusted for factors suspected to confound results, such as level and completeness of injury, age, and education.

## Discussion

Cognitive impairment is a multifaceted condition yet there is no research into its subtypes in people with an acute SCI. Different operationalizations of cognitive impairment pervade the SCI literature making it difficult to specify the extent and nature of the problem. Factors proposed to account for cognitive impairment in individuals with SCI include age at injury, autonomic dysfunction, cerebrovascular compromise, hypertension, hypotension, and psychosocial factors like depressive mood, chronic pain, and fatigue. It is possible that in some cases cognitive impairment can be associated with an iatrogenic consequence of medication-use, however, research into this hypothesis involving patients with SCI is sparse. Research is needed to identify all predictors of cognitive impairment following SCI, with specific attention given to subtyping. Studies that incorporate longitudinal designs and repeated measures permitting the identification of distinct trajectories and predictors of MCI are required. Therefore, this study will incorporate predictor and outcome measures in a prospective design beginning in the first 24-h of admission to the emergency department following SCI, the timeframe in which assessments of mild TBI must be made.

TBI is a common comorbidity of SCI that is assumed to be a significant contributor to cognitive impairment, and this must be carefully accounted for when investigating the unique contribution of other possible contributors to cognitive impairment. Various biopsychosocial measures (e.g., heart rate, mood, fatigue, and anxiety) will be included in the study, permitting the investigation of the additive and/or multiplicative risks that contribute to MCI. We propose the findings of this study will for the first time reveal important differences in the manifestation of cognitive impairment in individuals with SCI, and seek to develop evidence-based guidelines that will incorporate study findings to guide the rehabilitation of individuals with SCI who concurrently have MCI.

## Data Availability

All datasets used and/or analyzed during the current study are available from the corresponding author on reasonable request.
